# A harmonized dataset of education- and age-specific fertility rates for DHS countries

**DOI:** 10.1038/s41597-026-07136-z

**Published:** 2026-04-08

**Authors:** Afua Durowaa-Boateng, Dilek Yildiz, Anne Goujon

**Affiliations:** 1 Vienna Institute of Demography of the Austrian Academy of Sciences, Wittgenstein Centre for Demography and Global Human Capital (IIASA/POPJUS, OeAW/VID, Univ. Vienna/DoD), Vienna, Austria; 2 International Institute for Applied Systems Analysis, Wittgenstein Centre for Demography and Global Human Capital (IIASA/POPJUS, OeAW/VID, Univ. Vienna/DoD)of Vienna), Laxenburg, Austria

## Abstract

Education and fertility levels of individuals and communities are closely interlinked. Education-specific fertility rates are useful to understand how differences in educational attainment influence reproductive behaviour. These estimates also serve as foundations for designing and developing population projections under varying education and fertility scenarios. However, consistent series of historical education- and age-specific fertility rates are rarely available outside countries with strong vital registration systems. Here, we estimate education- and age-specific fertility rates for 78 countries within the Demographic and Health Surveys (DHS) database, spanning the period from 1970 to 2020, in five-year intervals. Using a Bayesian approach, we combine data from multiple sources to estimate UN-consistent education- and age-specific fertility rates.

## Background & Summary

Education plays a vital role in the demographic transition and particularly the transition from high to low fertility^1^. The role of education in accelerating this process encompasses increased autonomy^2^, access to and utilization of contraceptives, delayed childbearing that inadvertently lowers fertility levels, and the rising opportunity costs associated with expanded economic opportunities among others^3–8^. The reverse also holds, where declines in fertility rates at younger ages (especially adolescent and teenage pregnancies), foster and increase the chances of higher educational attainment^9,10^. Given the well-established relationship between education and female fertility, it is imperative to develop historically comparable fertility rates by educational attainment. These estimates would be useful, for instance, to track the impacts of past educational policies on fertility rates and project the future trajectories of both fertility rates and population sizes.

The age-specific fertility rate (ASFR) is calculated by dividing the number of live births to women in a specific age group during a given year by the total number of women in that same age group. The total fertility rate (TFR) is then calculated by summing the ASFRs across all reproductive ages (typically 15 to 49) and multiplying this sum by the width of the age interval (usually five years when ASFRs are grouped, or one year when using single-year ages). Data to calculate fertility rates are ideally obtained from complete vital registration systems, which provide reliable and annual data on births by the mother’s single years of age. However, such complete registration systems are still lacking in many Global South countries. Even in Global North countries with vital registrations, the data availability is often limited to the most recent decades. In the absence of a valid registration system, data from population and housing censuses and representative demographic surveys are used as alternative sources. Censuses collect detailed information on lifetime fertility and the date of the last child born alive^11^ but are prone to misreporting and underestimation of fertility rates over time. Moreover, not all countries—particularly Global South countries—conduct censuses regularly, which limits the availability and comparability of fertility data.

The Demographic and Health Surveys (DHS) are the main source of demographic data in low- and middle-income countries. Demographic and Health Surveys provide data for multiple surveys and consist of about 90 countries with surveys spanning from 1984 to 2025 (ongoing), with the most recent available surveys from 2023–2024^12^. The birth history module of the standard DHS collects retrospective information from the female respondents in reproductive age groups, including the details of their pregnancies, births, and children. The Multiple Indicator Cluster Surveys programme (MICS)^13^, by the United Nations Children’s Fund (UNICEF), provides data for over 120 countries since 1990. The survey modules, much like the DHS, comprise birth histories and other indicators of women^14^. The Malaria Indicators Survey (MIS), conducted in over 30 countries starting from the 2000s (and ongoing), also implemented by the DHS program, combines modules within the standard DHS and MICS^12^. For Europe, the Gender and Generation Survey (GGS), a cross-national panel survey focusing on the life-course and family dynamics, has been collecting data on fertility for more than 20 countries since 2004^15^.

Multiple global databases combine fertility data from vital registrations, censuses, and demographic surveys. However, no source provides harmonized data on education- and age-specific fertility for a large number of Global South countries spanning multiple decades. Historical ASFRs are more widely available as various international organisations collect and analyse such data. The United Nations World Population Prospects (UN WPP)^16^, produced by the UN Department of Economic and Social Affairs, provides the most widely used global and long-term series of demographic indicators, including TFRs and ASFRs. These indicators are derived from censuses, surveys, and vital registration systems, analysed vigorously and harmonized using Bayesian hierarchical models^17^. The UN WPPs are updated approximately every two years and offer data for 237 countries and areas from 1950. Complementary estimates are available from other UN bodies such as the UN Economic Commission for Latin America and the Caribbean (ECLAC)^18^, which publishes regional fertility data, together with additional indicators. For Europe, Eurostat, the statistical institute of the European Union, publishes age-specific fertility rates and number of live births by mothers’ age and educational attainment, as reported by the national statistical institutes of 46 countries for the 2007-2023 period^19^. The United States (US) Census Bureau International Database compiles and estimates TFRs and ASFRs as well as age-specific births for 227 countries and equivalent areas starting from 1950^20^.

In addition, several research institutes and research teams compile and harmonize historical fertility measures. At the global level, the Wittgenstein Centre for Demography and Global Human Capital (WIC) and the Institute for Health Metrics and Evaluation (IHME)^21^ provide historical population counts by age and sex, as well as demographic indicators, including fertility rates^22^. WIC’s historical population series is modelled on the UN WPP and disaggregated by educational attainment; however, its historical fertility estimates are not education-specific, even though WIC produces projections of education-specific fertility rates from 2020 until 2100. IHME’s historical fertility rates are estimated using mixed-effects regression models combined with spatiotemporal Gaussian process regression^23^. For developed countries, the Human Fertility Database^24^ provides historical and recent fertility data for 33 countries and areas with full coverage of vital statistics for different time periods, starting as early as the 1890s for Sweden^25^. Similar to the Human Fertility Database, the Cohort Fertility and Education (CFE) Database publishes data mostly for the European and developed countries in other regions. The CFE Database, being one of the few databases that provides education-specific fertility data, publishes completed cohort fertility and parity distribution by level of education for about 45 countries using data from 2001 to 2011 censuses^26^. For Global South countries, the African Fertility Data Explorer (AFD)^27^ provides harmonized education-specific fertility rates from 1970 to 2020 for 41 countries in Africa. In this paper, we build on the methodology of AFD using Bayesian models and extend the education-specific ASFR (EAFR) and TFR (ETFR) data set to 78 countries for which DHS survey data are available^6,7^.

Several studies and reports have found that the quality of DHS surveys varies over time and across countries due to recall error, age heaping, omission of events, and sampling error^28,29^. To address these challenges and borrow information from multiple data sources, Bayesian models have been employed for demographic estimation, particularly for fertility, mortality, and migration when registration systems are incomplete. At the global scale, Bayesian hierarchical models are used in the United Nations probabilistic population projections and in their under-five mortality estimations^30–32^. At the national, sub-national, and regional levels, several studies have applied Bayesian models and other methodologies to estimate both education-specific and overall mortality, fertility, and migration rates^6,7,33–43^.

In the remainder of this article, we first outline our methodology and highlight the differences with the previous studies. We illustrate education-specific ASFRs and TFRs over time. We then provide some guidance for potential users of the dataset and present the results of technical validation.

## Methods

### Data Sources

To estimate five-year *EAFR* and *ETFR* for 78 countries between 1970–1975 and 2015–2020 period, we obtained and cleaned data from the DHS, the UN WPP 2022, and the Wittgenstein Centre Human Capital Data Explorer (WIC)^12,17,44^. Although the UN WPPs have a 2024 round of projections, this study employs the 2022 rounds to ensure consistency across the input data. The WIC education estimates are calibrated to the UN WPP 2022 rounds of revision. Updating the benchmark from the 2022 to the 2024 revision would change the final estimates, because the model benchmarks the overall ASFR schedule to the UN WPP. The *EAFR* estimates are for five-year groups from 15-19 to 45-49, spanning the standard reproductive ages for women. In both *EAFR* and *ETFR*, education represents the highest educational attainment category for the respondent, as indicated by the DHS^45^. The categories are “No Education”, “Primary Education”, “Secondary Education” and “Higher Education”.

All input data are publicly available and freely downloadable; however, the DHS microdata require free registration. The UN WPP fertility estimates can be accessed on the UN WPP site at https://population.un.org/wpp/downloads?folder=Standard%20Projections&group=Fertility. The education-specific birth histories from the DHS microdata can be accessed free of charge after user registration with the DHS program at https://www.dhsprogram.com/data/new-user-registration.cfm^12^. To derive the estimates for the GLM in Equation 1 below, input data are obtained using the Individual/women’s recode (IR) file. The recode files are processed in STATA using the “tfr2” module and follow the example code structure below:


use CCIR01FL.DTAby v106, sort: tabexp, len(30) trend(5) ageg(5) cy rates


The above code, which is an example replica for the estimates used, generates the birth histories for the 30 years before the survey from the survey wave by level of education. The file name above is an example of the general file name of the DHS data, which is country code or abbreviation (CC), file type, in this case individual recode (IR), survey wave (usually numeric), followed by FL.DTA. The study draws on data from 332 DHS surveys, incorporating all available survey waves up to 2024^12^. This process is repeated for all countries and survey waves listed in Table 1 (343 waves go into the GLM, however, 11 survey waves for Nepal, Uzbekistan, Ukraine, Afghanistan, Papua New Guinea and Mauritania are not included in the final Bayesian model due to very poor data quality). The resulting data is cleaned in R, where the reported birth count exceeding 30 is removed for women who have higher education and other implausible point estimates after visualizing the data. The cleaned data form the basis of the GLM, and the predicted estimates are saved as the initial values for the Bayesian model.

**Table 1:** Country list with survey years.

Survey Year	Countries
1985	El Salvador
1986	Brazil, Colombia, Dominican Republic, Liberia, Peru, Senegal
1987	Burundi, Ecuador, Guatemala, Indonesia, Mali, Mexico, Morocco, Sri Lanka, Thailand, Trinidad and Tobago
1988	Egypt, Ghana, Kenya, Togo, Tunisia, Uganda, Zimbabwe
1989	Bolivia, Sudan
1990	Jordan, Nigeria, Pakistan, Paraguay
1991	Brazil, Cameroon, Dominican Republic, Indonesia, Peru, Tanzania, Yemen
1992	Burkina Faso, Egypt, India, Madagascar, Malawi, Morocco, Namibia, Niger, Rwanda, Senegal, Zambia
1993	Bangladesh, Bolivia, Ghana, Kenya, Philippines, Turkey
1994	Central African Republic, Côte d’Ivoire, Haiti, Indonesia, Zimbabwe
1995	Colombia, Egypt, Guatemala, Kazakhstan, Mali, Uganda
1996	Bangladesh, Benin, Brazil, Chad, Comoros, Dominican Republic, Peru, Tanzania, Zambia
1997	Indonesia, Jordan, Kyrgyzstan, Madagascar, Mozambique, Nicaragua, Senegal, Vietnam
1998	Bolivia, Burkina Faso, Cameroon, Côte d’Ivoire, Ghana, Guatemala, India, Kenya, Niger, Philippines, South Africa, Togo, Turkey
1999	Bangladesh, Dominican Republic, Guinea, Kazakhstan, Mozambique, Tanzania, Tunisia, Zimbabwe
2000	Armenia, Cambodia, Colombia, Egypt, Ethiopia, Gabon, Haiti, Malawi, Namibia, Peru, Rwanda, Uganda
2001	Benin, Mali, Nicaragua, Zambia
2002	Dominican Republic, Indonesia, Jordan, Vietnam
2003	Bolivia, Burkina Faso, Egypt, Ghana, Kenya, Madagascar, Morocco, Mozambique, Nigeria, Peru, Philippines, Turkey
2004	Bangladesh, Cameroon, Chad, Colombia, Lesotho, Malawi, Tanzania
2005	Armenia, Cambodia, Congo, Egypt, Ethiopia, Guinea, Guyana, Haiti, Honduras, India, Moldova, Rwanda, Senegal, Vietnam, Zimbabwe
2006	Angola, Azerbaijan, Benin, Eswatini, Liberia, Mali, Namibia, Niger, Pakistan, Uganda
2007	Bangladesh, Democratic Republic of Congo, Indonesia, Jordan, Rwanda, Zambia
2008	Albania, Bolivia, Egypt, Ghana, Kenya, Liberia, Madagascar, Nigeria, Philippines, Sao Tome and Principe, Sierra Leone, Turkey
2009	Colombia, Guyana, Jordan, Lesotho, Maldives, Peru, Tanzania, Timor-Leste, Uganda
2010	Armenia, Burkina Faso, Burundi, Cambodia, Malawi, Nigeria, Peru, Rwanda, Senegal, Zimbabwe
2011	Angola, Bangladesh, Benin, Cameroon, Congo, Côte d’Ivoire, Ethiopia, Honduras, Liberia, Madagascar, Mozambique, Peru, Uganda
2012	Burundi, Comoros, Gabon, Guinea, Haiti, Indonesia, Jordan, Kyrgyzstan, Malawi, Mali, Niger, Pakistan, Peru, Senegal, Tajikistan
2013	Democratic Republic of Congo, Gambia, Guatemala, Liberia, Madagascar, Namibia, Nigeria, Philippines, Rwanda, Sierra Leone, Togo, Turkey, Yemen, Zambia
2014	Bangladesh, Burkina Faso, Cambodia, Chad, Egypt, Ghana, Kenya, Lesotho, Malawi, Rwanda, Senegal, Uganda
2015	Angola, Armenia, Colombia, India, Kenya, Malawi, Mali, Mozambique, Myanmar, Nigeria, Senegal, Tanzania, Zimbabwe
2016	Burundi, Ethiopia, Ghana, Haiti, Liberia, Madagascar, Maldives, Senegal, Sierra Leone, South Africa, Timor-Leste, Uganda
2017	Albania, Bangladesh, Benin, Burkina Faso, Indonesia, Jordan, Pakistan, Philippines, Rwanda, Senegal, Tajikistan, Tanzania, Togo
2018	Cameroon, Guinea, Mali, Nigeria, Senegal, Uganda, Zambia
2019	Ethiopia, Gambia, Ghana, Liberia, Senegal, Sierra Leone, Turkey
2020	Rwanda
2021	Burkina Faso, Côte d’Ivoire, Gabon, Guinea, India, Madagascar, Mali, Niger, Nigeria
2022	Bangladesh, Cambodia, Cameroon, Kenya, Liberia, Philippines, Tanzania
2023	Ghana, Jordan, Mozambique, Senegal
2024	Lesotho

Education-specific population sizes used in the Bayesian model are collected from the Wittgenstein Centre for Demography and Global Human Capital using the R package wcde, specifically the 2018 WIC estimates denoted by wcde-v2 in the package^44^.

For sub-Saharan African countries, previous estimates of Bayesian education-specific fertility rates were collected. The UN-consistent Bayesian education-specific total fertility rates for sub-Saharan African countries (ETFR DHS UN) can be publicly downloaded at Zenodo (10.5281/zenodo.18185201)^46^.

### Model structure

The main aim of the proposed methodology is to disaggregate total and age-specific fertility rates by level of education. The approach relies on a single source for overall fertility rates (the UN WPP), a primary source for education-specific fertility rates (the DHS), and a source for population size by level of education (the WIC). Additionally, education-specific fertility estimates from a previous harmonisation effort were included in the modelling framework for consistency^7^.

Following Durowaa-Boateng *et al*.^6^, we employ a two-step approach. In the first step, initial EAFRs are generated using a Generalized Linear Model (GLM) to complete age schedules that are only partially observed in the DHS microdata. These estimates are then used as initial values in the second step, where they inform the hierarchical Bayesian model. The main difference between our model and previous research is the joint estimation of fertility rates. This improvement allows the model to share information across regions, rather than modeling them separately. The region parameter follows the UN regions: sub-Saharan Africa, North Africa, West Asia, Europe, Central Asia, South & Southeast Asia, and Latin America.

### Step 1: Modelling initial values from DHS (GLM)

To estimate the initial values, we use a generalized linear model (GLM) following the equation,

1 $$\begin{array}{rcl}EAF{R}^{DHS} &  \sim  & Education+Region+Age\,Group+Country+Year\\  &  & +Education\cdot Age\,Group+Year\cdot Age\,Group\\  &  & +Region\cdot Age\,Group+Region\cdot Year+Region\cdot Country.\end{array}$$

In Equation 1, *EAFR*^*DHS*^ represents the education- and age-specific fertility rates from the Demographic and Health Surveys. Due to the joint estimation across regions, a *Region* parameter is included in the GLM, which was not considered in Durowaa-Boateng *et al*.^6^.

### Step 2: Three-level Bayesian Hierarchical Model

**Fig. 1:**
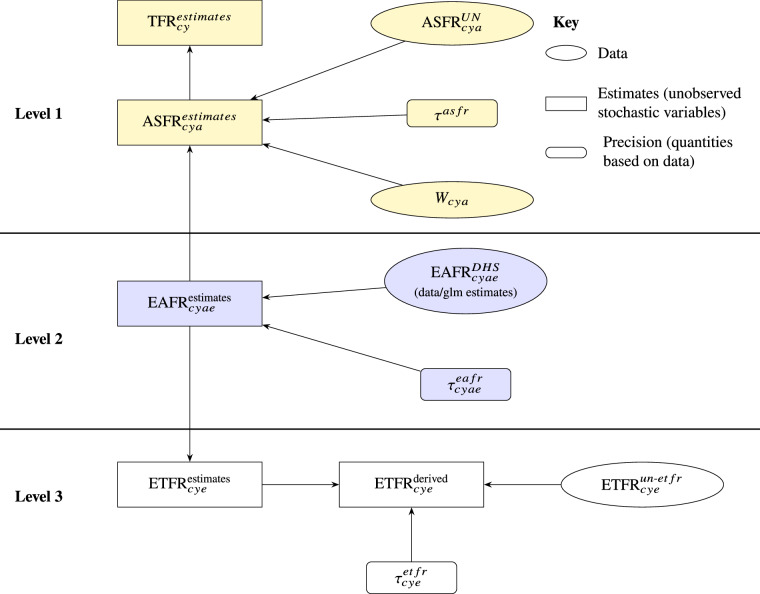
Graphical presentation of the Bayesian model framework.

The second step implements a three-level hierarchical Bayesian framework, as shown in Fig. 1. In this step, in addition to the primary data sources, we also include previously published fertility estimates by education level for DHS countries in the Bayesian model. This helps ensure consistency for countries that were part of earlier harmonisation efforts.

Level 1 benchmarks age-specific fertility rates to the UN WPP ASFRs, Level 2 estimates EAFR, and Level 3 produces ETFR. The fertility estimates are for each country *c*, five-year period *y*, education level *e* and five-year age-group *a*, where necessary. These categories remain similar to those in the GLM in Equation 1.

### Harmonizing education-specific fertility rates

Level 1 of the framework ensures the consistency of the estimated *EAFR* values with the UN WPP^17^*ASFR*. By applying education- and age-specific female population weights (WIC pop data) to the rates, this level ensures that the weighted education-specific fertility rates are in alignment with the UN WPP’s age-specific fertility rates.

The mathematical formulations of Level 1 in Fig. 1 follow:

2 $$TF{R}_{cy}^{estimates}=\mathop{\sum }\limits_{a=15-19}^{45-49}\left(ASF{R}_{cya}^{estimates}\right)\cdot 5$$

3 $$ASF{R}_{cya}^{UN} \sim {N}_{+}\left(ASF{R}_{cya}^{estimates},{\tau }^{asfr}\right)$$

4 $$ASF{R}_{cya}^{estimates}=\mathop{\sum }\limits_{e=1}^{4}\left(EAF{R}_{cyae}^{estimates}\cdot {w}_{cyae}\right)$$

5 $${\tau }^{asfr}=1/{\sigma }^{asf{r}^{2}}$$

Quantities with the superscript “estimates” are the unknown parameters that are estimated within the posterior sampling. The variable *ASFR*^*UN*^ is the age-specific fertility rate from the UN WPP. The parameter ${\sigma }^{asf{r}^{2}}$ is the variance of the UN WPP’s age-specific fertility rates. *τ*^*asfr*^ represents the precision of the ASFR estimates in Equation 3. The variable *w*_*cyae*_ denotes the weights derived from the WIC population by education level.

The output of the GLM model ($EAF{R}_{cyae}^{DHS}$ (data/glm estimates)) enters the modelling framework in Level 2 as initial values to estimate $EAF{R}_{cyae}^{estimates}$. As shown in Equation 6, the EAFRs are modelled using a truncated normal distribution. The distribution is centred on the GLM estimates ($EAF{R}_{cyae}^{DHS}$) generated in Equation 1. The quantity ${\sigma }_{e}^{eafr}$ represents the education-specific standard deviations of the standard errors derived from the GLM model. The equations for Level 2 are:

6 $$EAF{R}_{cyae}^{estimates} \sim {N}_{+}(EAF{R}_{cyae}^{DHS},{\tau }_{cyae}^{ea\,fr}),$$

7 $${\tau }_{cyae}^{ea\,fr} \sim G(1/{\sigma }_{e}^{ea\,fr},\,2{\sigma }_{e}^{ea\,fr})$$

Finally, Level 3 follows:

8 $$ETF{R}_{cye}^{derived}=\left(\mathop{\sum }\limits_{a=15-19}^{45-49}EAF{R}_{cyae}^{estimates}\right)\cdot 5$$

9 $$ETF{R}_{cye}^{estimates} \sim \left\{\begin{array}{ll}{N}_{+(0,10)}(ETF{R}_{cye}^{derived},{\mu }_{cye}^{et\,fr}), & \,{\rm{for\; all\; regions}}\\ ETF{R}_{cye}^{un-et\,fr} \sim {N}_{+(0,10)}(ETF{R}_{cye}^{derived},{\mu }_{cye}^{et\,fr}), & \,{\rm{for\; sub\; -\; Saharan\; Africa}}\\ \end{array}\right.$$

10 $${\tau }_{cye}^{et\,fr} \sim G(1/{\sigma }_{e4}^{et\,fr},2\cdot {\sigma }_{e4}^{et\,f{r}^{2}})$$

The quantities *σ*^*etfr*^ and ${\sigma }^{etf{r}^{2}}$ are the standard deviation and variance, respectively, of the education-specific total fertility rates for the highest education level (Higher Education). In level 3, the estimates only for the sub-Saharan African countries are in place to ensure some degree of consistency between the education-specific TFR estimates in Yildiz *et al*.^7^, which were also used as a benchmark in Durowaa-Boateng *et al*.^6^ and the current estimates.

## Data Overview

**Fig. 2:**
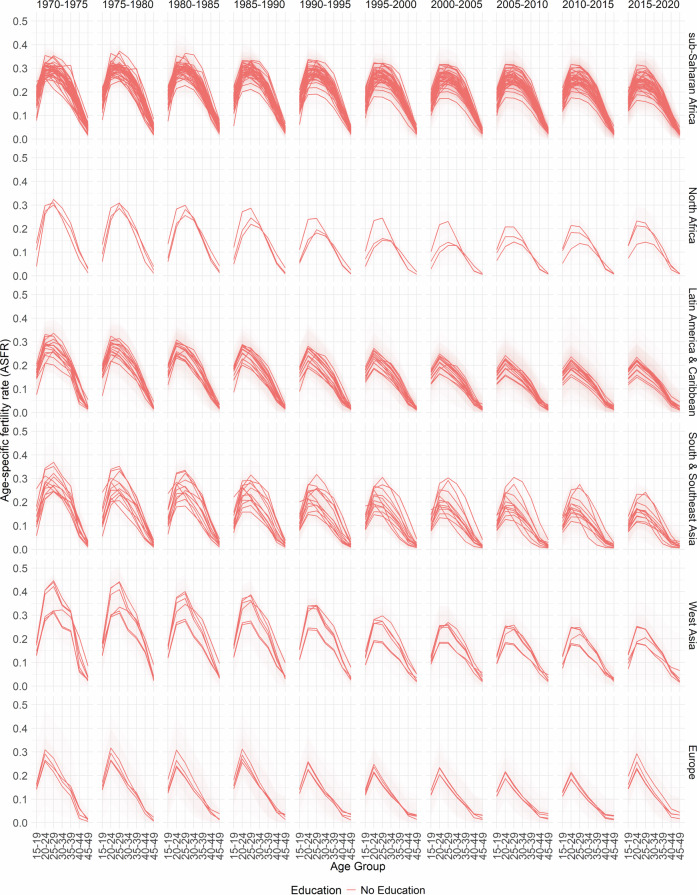
No Education- and age-specific fertility rates.

**Fig. 3:**
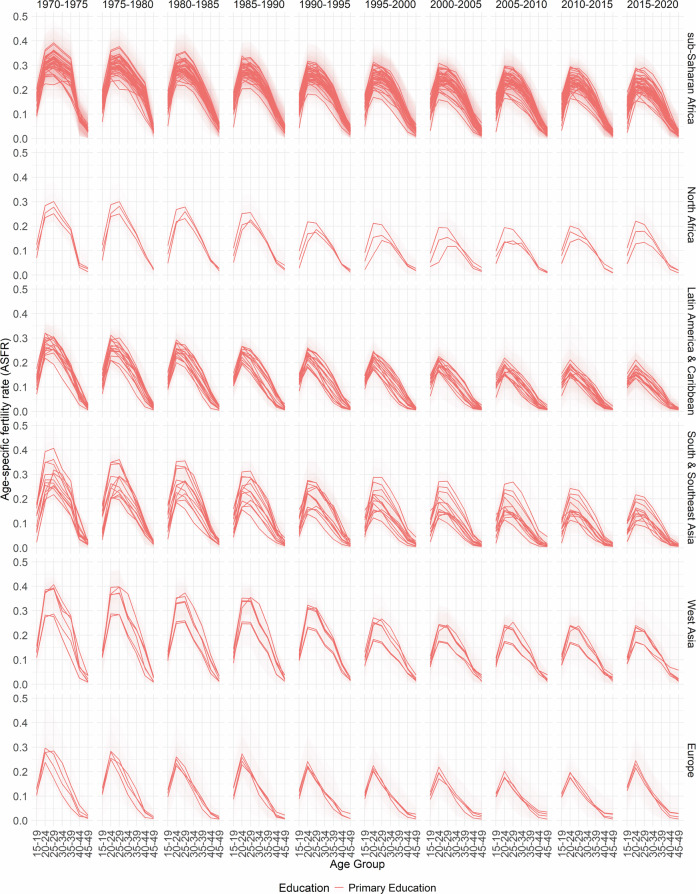
Primary Education- and age-specific fertility rates.

**Fig. 4:**
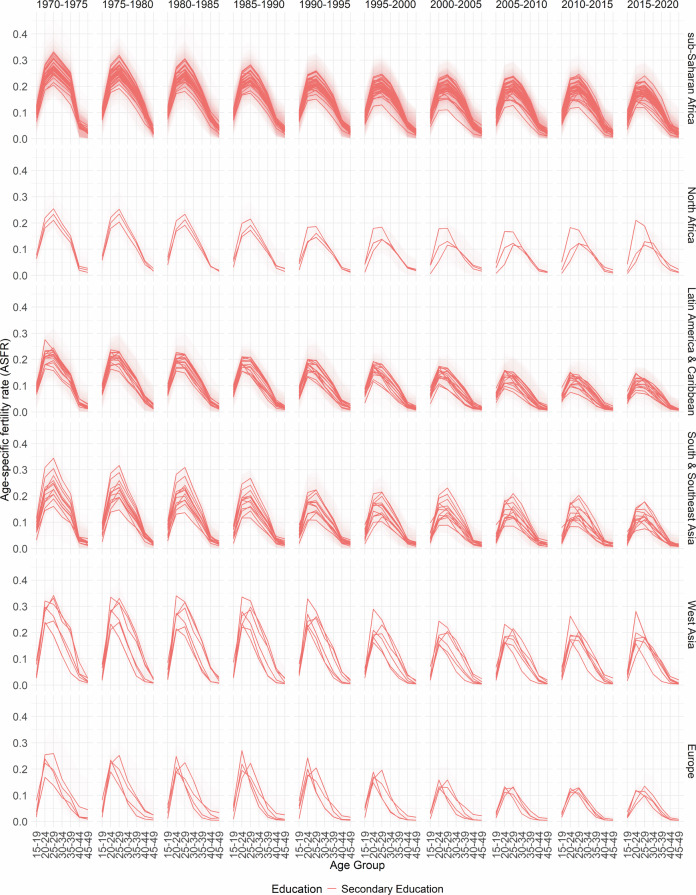
Secondary Education- and age-specific fertility rates.

**Fig. 5:**
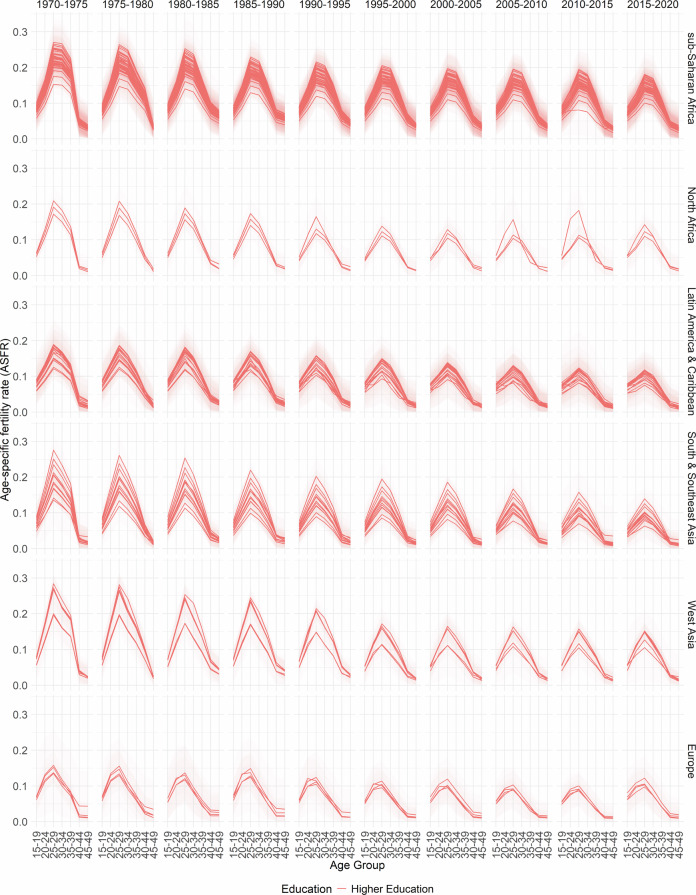
Higher Education- and age-specific fertility rates.

**Fig. 6:**
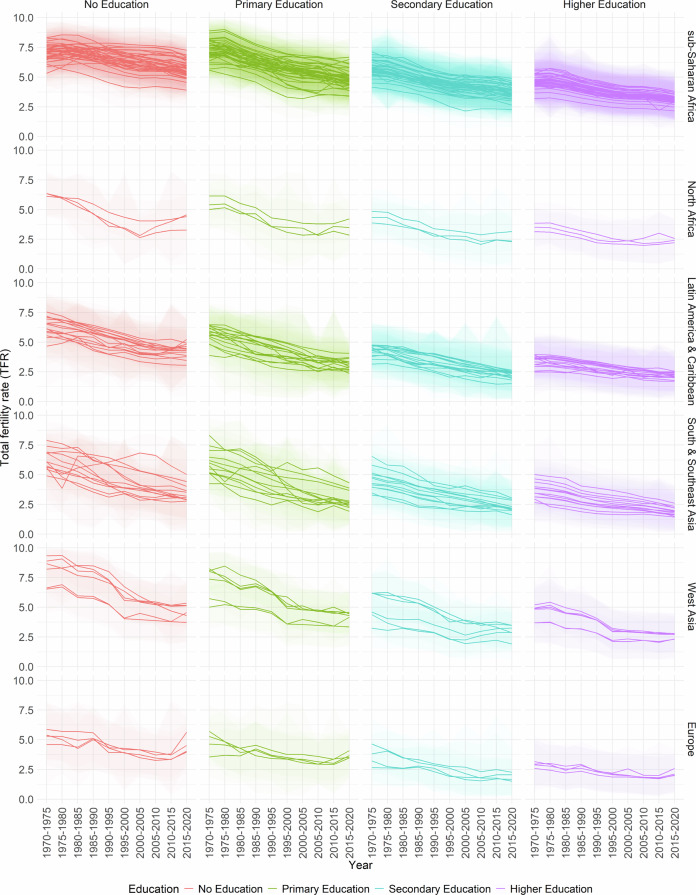
Education-specific total rates.

In Figs. 2, 3, 4, 5 and 6, the median estimates of the EAFRs (posterior estimates) for all DHS countries are presented together with 95% credible intervals (CI) and grouped by sub-regions for each education level. These results point to an overall decline in EAFRs and ETFRs across all countries and education levels. Across all regions, estimates for the education levels “Secondary Education” and “Higher Education” are generally lower than “No Education” and “Primary Education”.

The estimates, in terms of the curvature of age-specific fertility rates, show a well-documented shift in age schedules both across the years and education levels (postponement)^3–5^. The results and estimated patterns also show that in some countries, particularly in sub-Saharan Africa, primary-education-specific fertility rates were higher than those for women with no education in the earlier years (1970–1975). This phenomenon has been previously established and observed in other studies for that period, as women moved away from traditional birth-spacing practices (shortening postpartum abstinence and relying more on bottle feeding/shorter breastfeeding), thereby reducing the postpartum non-susceptible period and increasing the pace of childbearing relative to women with no education^8,47,48^.

Furthermore, it is well documented that conflicts influence fertility rates at different times. In areas of conflict or natural disasters, the immediate response is usually high mortality and subsequently declining fertility rates; however, periods succeeding these periods are marked by high fertility rates^49,50^. While the effects of conflicts on fertility rates are observed in the TFRs, they influence the timing of fertility (ASFRs). There are various mechanisms through which conflicts, as well as other events, affect the ages at which women have children. These events could directly or indirectly cause postponement among age groups—particularly in periods of active conflicts—thus leading to shifts in the age-specific fertility curve and overall TFR^51–54^.

It is important to note that the fertility schedules observed in different regions and countries may have different reasons, and those listed here are not the sole reasons for the shape of the fertility curve. For some periods, particularly the 1970s in sub-Saharan Africa, the nature of the fertility curve and overall fertility rates could be due to a variety of events, including conflicts, such as early and near universal marriages for women, reduced female education, which has been linked to lower fertility rates and postponements, polygamy and a high desired number of children among others^55^. Similarly, each country has its own age at which fertility rates are highest. This happens in different years at different age groups. The country-specific plots can be found on the GitHub repository https://github.com/AfuaD-B/Bayesian-methodology-on-fertility-reconstruction.

In Fig. 6, the estimates reflect period fluctuations observed in the individual countries. For instance, the distinct dip detected in the ETFR of no and primary education in Cambodia from 1975 to 1980 is indicative of the actual overall TFR of the country and has been recorded in other studies^56,57^. The cause of the sharp decline in Cambodia has in part been due to the political events during the “Khmer Rouge” regime^58^. In addition to Cambodia in South and Southeast Asia, Timor-Leste’s fertility pattern is attributed to previous years of conflicts^59^. Generally, women with no and primary education in lower-educated populations drive overall fertility rates due to their larger share in the composition of the female populations^60^. As with the case of Cambodia, the model output reflects patterns consistent with events and other contextual factors that may have influenced fertility rates at that time.

## Data Records

The dataset containing the education-specific estimates for the five-year age groups from 1970 to 2020 is available at Zenodo (10.5281/zenodo.18359482)^61^ and mirrored on GitHub under the repository https://github.com/AfuaD-B/Bayesian-methodology-on-fertility-reconstruction. The Zenodo contains the main dataset generated from this model, “BESFR_estimates global_south.xlsx”. This Excel workbook has the median estimates together with the credible intervals of the education-specific fertility estimates. The file contains the columns “Country”, “Age Group”, “Education”, “Year”, “Upper_CI”, “Lower_CI”, and “Median”. The columns “Upper_CI”, “Lower_CI” represent the 95% credible intervals. Only in the GitHub repository are the separate country-specific EAFRs provided in a PDF titled “final results_plot.pdf”. The Zenodo record also contains additional output files, including the Excel workbooks “edu_spec_sd_init_prior.xlsx”, which contains the Bayesian priors from the main model, and “edu_diff.xlsx”, which contains the estimated differences in education- and age-specific fertility rates across education levels. Code files used to fit the Bayesian models, generate the estimates and figures, and run the sensitivity and validation analyses are available in both Zenodo and GitHub, and are described in the Code Availability section.

## Technical Validation

**Fig. 7:**
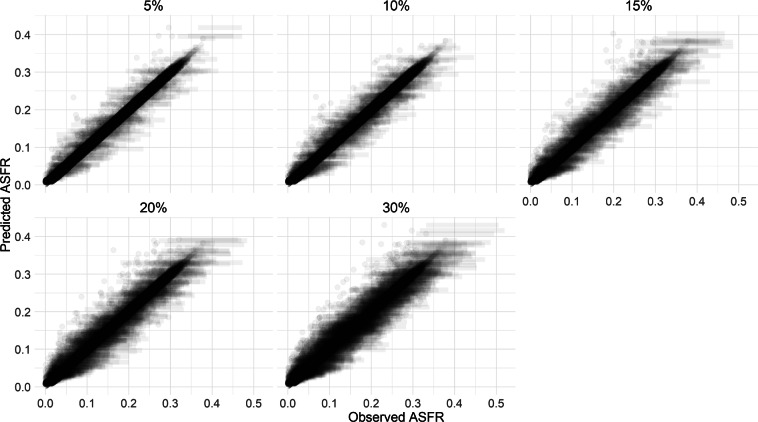
Model validation results of omitted percentages.

**Fig. 8:**
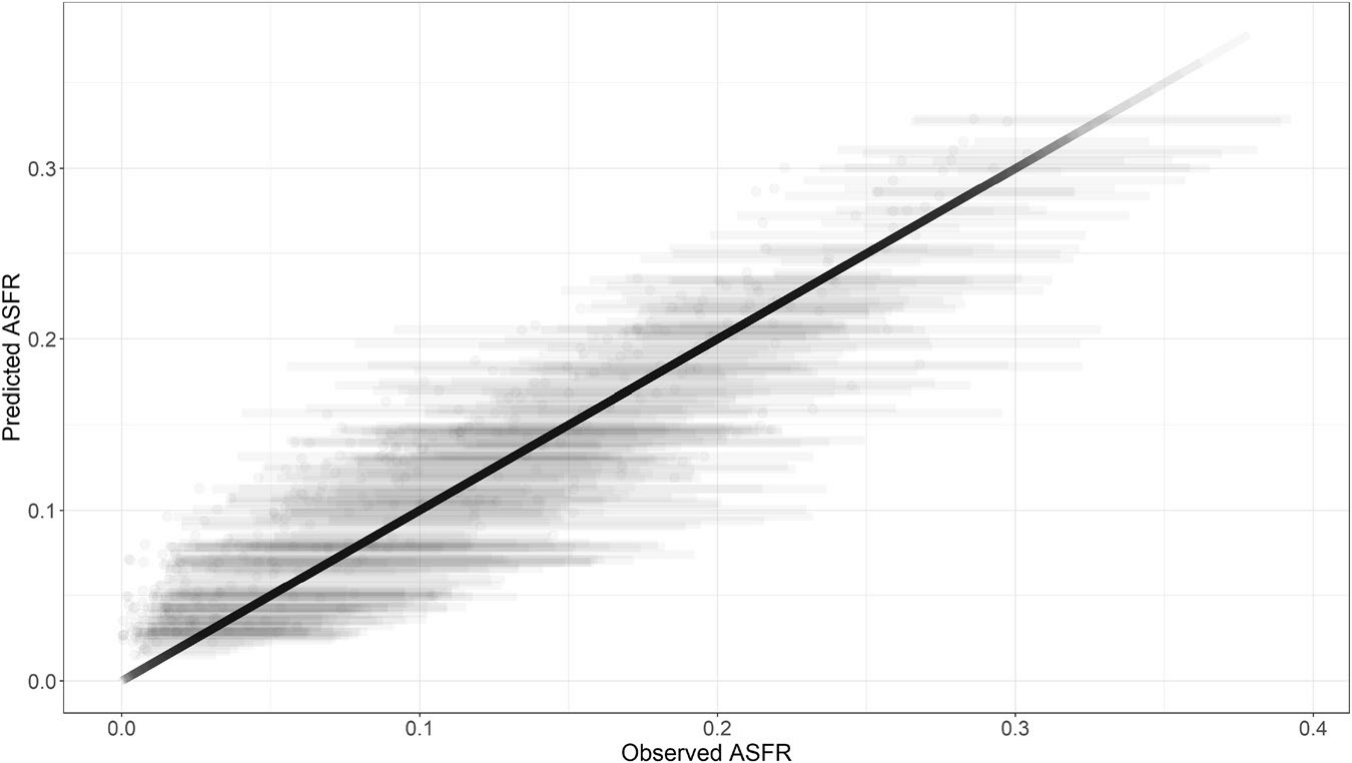
Model validation results of omitted countries.

In this model estimation, we ran two validation models, both of which are random holdout validation approaches. In the first approach, 5%, 10%, 15%, 20%, and 30% of the UN *ASFR* estimates were randomly left out of the model (Fig. 7). We then refit the model for the remainder of the dataset. The second version omits data from five countries: Albania, Bangladesh, Colombia, Ghana, and the Philippines (Fig. 8). The data were randomly removed only from the UN WPP’s ASFR. Since the model is benchmarked to the UN ASFRs, we removed some UN ASFR values and refit the model to see how well it can recover the missing benchmark values from the remaining ones. This tests how sensitive the model is to missing values in the UN benchmark ASFR schedule.

Table 2 shows the percentages of observations that fall within 50%, 80%, and 90% credible intervals by the country omitted.

**Table 2:** Credible interval coverage of validation by Country.

Country omitted	50% CI	80% CI	90% CI
Albania	66.0%	82.2%	87.3%
Bangladesh	66.0%	82.1%	87.1%
Colombia	66.0%	81.9%	86.9%
Ghana	66.4%	82.5%	87.2%
Philippines	66.4%	82.4%	87.2%

Similarly, Table 3 shows the coverage by percentages omitted.

**Table 3:** Credible interval coverage of validation by percentages omitted.

Percentage omitted	50% CI	80% CI	90% CI
5%	64.6%	80.9%	86.2%
10%	63.0%	79.8%	84.9%
15%	61.0%	78.2%	83.7%
20%	60.2%	76.8%	82.7%
30%	55.8%	73.6%	80.4%

In our results, we find that the credible interval coverage declines as more benchmark data are removed, which is expected because the model has less information to anchor the age pattern. When only a small share of the benchmark is removed (for instance, 5–10%), the 80% intervals contain about 80% of the held-out values. When a larger share is removed (30% in this case), coverage falls, particularly, the 90% credible intervals contain fewer than 90% of the held-out values in these tests (about 80–87% across Tables 2 to 3). These validation results imply that the model becomes less precise when the benchmark is sparse. These checks suggest the model can reproduce the UN benchmark ASFR schedule reasonably well when some benchmark values are missing, but it becomes less precise as more benchmark data are removed. Due to the unavailability of education-specific fertility rates for all the countries estimated, the validation of the EAFRs themselves was not conducted.

Additionally, we ran sensitivity analyses using different priors. These codes are available in the GitHub repository. The sensitivity analysis comprised seven different prior models for the precision parameter ${\tau }_{cyae}^{eafr}$. Table 4 shows the different prior distributions used in the sensitivity analysis.

**Table 4:** Different prior distributions for EAFR precision.

Model	Distribution	Source
Final model	${\tau }_{cyae}^{ea\,fr} \sim G(1/{\sigma }_{e}^{ea\,fr},\,2{\sigma }_{e}^{ea\,fr})$	standard deviations of standard errors by education level (GLM)
Model 1	*τ* ~ *G*(*α*, *β*)	relative variance, variance-mean ratio of standard errors (GLM)
Model 2	$\tau \sim G(1/{\mu }_{e}^{ea\,fr},\,{\sigma }_{e}^{ea\,f{r}^{2}})$	mean and variance of standard errors by education level (GLM)
Model 3	$\tau \sim G(1/{\sigma }_{e}^{ea\,f{r}^{2}},\,{\sigma }_{e}^{ea\,f{r}^{2}})$	variance of standard errors by education level (GLM)
Model 4	$\tau \sim G(1/{\sigma }^{eaf{r}^{2}},\,{\sigma }^{eaf{r}^{2}})$	variance of standard errors (GLM)
Model 5	$\tau \sim G(1/{\sigma }^{eafr},\,{\sigma }^{eaf{r}^{2}})$	variance of estimates (GLM)
Model 6	${\tau }_{cyae}^{ea\,fr} \sim G(1/{\sigma }_{a,e}^{ea\,fr},\,{\sigma }_{a,e}^{ea\,fr})$	variance of standard errors by age and education level (GLM)
Model 7	${\tau }_{cyae}^{ea\,fr} \sim G(1/{\sigma }_{e}^{ea\,fr},\,{\sigma }_{e}^{ea\,fr})$	variance of standard deviation by education level (GLM)

We selected the final model because its estimated ASFRs align most closely with the UN WPP ASFRs among the prior specifications considered.

## Usage Notes

The methodology described in this article is flexible and adaptable to reconstruct or estimate education-specific rates, or fertility rates in general, in regions or areas where data are sparse and/or come from multiple data sources, not limited to the DHS. For future studies, the estimates generated from this methodology can be downloaded freely for population projections by level of education, policy evaluation, and other such studies. While the study employed the 2022 revision rounds of the UN WPP, a comparison between the Bayesian ASFRs from this exercise and the 2024 rounds showed that about 85% of the UN WPP 2024 ASFR values (country-period-age combinations) fell within the 95% credible intervals of the current model estimates. In addition, 47.1% of the ASFR values fall within the 50% CI, 72.1% within the 80% CI, and 80.4% fell within the 90% CI.

## Data Availability

The resulting outputs can be accessed on Zenodo (10.5281/zenodo.18359482) and GitHub at https://github.com/AfuaD-B/Bayesian-methodology-on-fertility-reconstruction in the Excel workbook “BESFR_estimates global_south.xlsx”. All other input data sources used to generate these outputs are described in the Methods section.

## Code availability

The codes used to fit the Bayesian models, run sensitivity and validation analyses, and generate the output files and figures are available at Zenodo (10.5281/zenodo.18359482)^61^ and mirrored on GitHub at https://github.com/AfuaD-B/Bayesian-methodology-on-fertility-reconstruction. All codes except the “tfr2” STATA module were run in RStudio software. The code for the final Bayesian model is in the file “Bayesian model global_south.R”. The validation code files are “Bayesian model global_south_validation country omission.R” and “Bayesian model global_south_validation.R”. The other models for the sensitivity analysis are in the code files, “glm input data global south model_alpha_beta.R” (Model 1 in Table 4), “glm input data global south model_edu_spec_mean.R” (Model 2 in Table 4), “glm input data global south model_edu_spec_sd.R” (Model 3 in Table 4), “glm input data global south model_gamma_47.R” (Model 4 in Table 4), “glm input data global south model_pred_prec.R” (Model 5 in Table 4), “glm input data global south model_se_sd_c_y_a.R” (Model 6 in Table 4) and “glm input data global south model_se_sd_c_y_e.R” (Model 7 in Table 4).
